# Synthesis of α‐Aryl Acrylamides via Lewis‐Base‐Mediated Aryl/Hydrogen Exchange

**DOI:** 10.1002/anie.202207475

**Published:** 2022-08-29

**Authors:** Miran Lemmerer, Haoqi Zhang, Anthony J. Fernandes, Tobias Fischer, Marianne Mießkes, Yi Xiao, Nuno Maulide

**Affiliations:** ^1^ Faculty of Chemistry Institute of Organic Chemistry University of Vienna Währinger Str. 38 1090 Vienna Austria; ^2^ Christian-Doppler Laboratory for Entropy-Oriented Drug Design Josef-Holaubek-Platz 2 1090 Vienna Austria; ^3^ Boehringer-Ingelheim RCV 1120 Vienna Austria; ^4^ CeMM Research Center for Molecular Medicine of the Austrian Academy of Sciences Lazarettgasse 14, AKH BT 25.3 1090 Vienna Austria

**Keywords:** Amides, Aromatic Substitution, C−C Coupling, Organocatalysis, Rearrangement

## Abstract

Herein we report a method for the synthesis of α‐aryl acrylamides leveraging polar *S*‐to*‐C* aryl migrations induced by a Lewis basic organocatalyst. In contrast to previously reported radical aryl migrations of sulfonyl acrylimides, this polar process enables subsequent elimination, ultimately leading to a formal aryl/hydrogen exchange including SO_2_ extrusion. This reaction is selective for electron‐deficient aromatic groups, while tolerating a variety of substituents on nitrogen and in the β‐position, and it delivers useful building blocks for further transformations, including cycloaddition and cyclisation reactions. The mechanism was investigated in detail using quantum chemical calculations, which unexpectedly revealed the Lewis base to be involved in several decisive steps.

## Graphical abstract

A DABCO‐induced polar aryl migration allows for the synthesis of α‐aryl acrylamides from sulfonyl acrylimides. This reaction proceeds through a formal aryl/hydrogen exchange, including SO_2_ loss. A range of valuable building blocks have been synthesised and the underlying reaction mechanism has been explored using quantum chemical calculations.

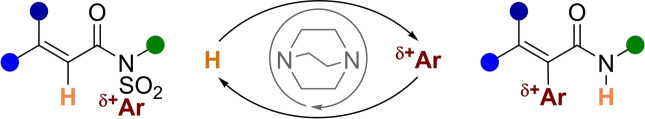

The dual‐functionality of α,β‐unsaturatedα‐arylcarboxamides renders them versatile building blocks for organic synthesis. In particular, the electron‐poor alkene moiety serves as a handle for their incorporation into structures of different applications and ring sizes (Scheme 1A).^[1]^ Interestingly, when one considers their synthesis, transition metal catalysis typically delivers the “wrong” regioselectivity; indeed, the formal α‐C−H arylation of secondary acrylamides has not been achieved, as traditional Heck‐type chemistry leads to β‐arylation (Scheme 1B).^[2]^ Cross‐coupling chemistry instead relies on either prior α‐functionalisation with halides (Scheme 1C)^[1e]^ or pre‐installed directing groups.^[3]^

**Scheme 1:**
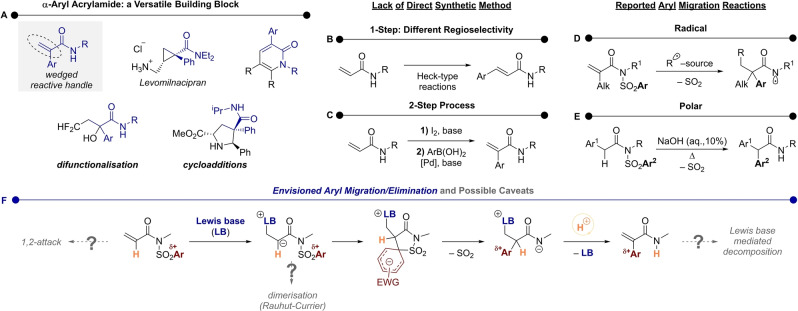
A) Examples of α‐aryl acrylamides used in cycloadditions and difunctionalisation. B) Heck‐type reactions lead to β‐arylation. C) Formal C−H‐α‐arylation requires a 2‐step process. D) Reports of α‐arylation via radical migration delivering saturated amides. E) A polar aryl migration of arylacetyl sulfonylimides. F) Our envisioned, Lewis‐base‐induced aryl migration followed by elimination. Possible caveats and side reactions are showcased.

In the past, aryl migration has shown to be a powerful tool for the synthesis of *saturated* α‐aryl amides.[^4^, ^5^] In a series of seminal publications, the Nevado group has used such a strategy on *N*‐sulfonyl *N*‐aryl methacrylimides (Scheme 1D).^[6]^ Therein, radical addition generates an α‐radical, which then engages in aryl migration (Truce‐Smiles rearrangement), ultimately leading to SO_2_ extrusion and formation of an *N*‐centred radical.^[7]^ Most recently, the group has reported an enantiospecific variant of this chemistry,^[8]^ while other groups have expanded this reactivity even further with a range of radical sources.[^9^, ^10^]

The strength of most reported methods lies in the migration of electron‐rich or ‐neutral aryl groups, with a dearth of reported examples pertaining to rearrangements of electron‐deficient aryls. Additionally, an alkyl group in α‐position seems to be required, with only a handful of α‐H acryl imides being reported.[^9b^, ^9h^, ^10^]

Drawing inspiration from reports by Dohmori^[11]^ and Greaney (Scheme 1E),^[12]^ we speculated that an *anionic* aryl migration might be ideally suited to prepare α‐aryl acrylamides (Scheme 1F). Concretely, we hypothesised that a Lewis base might initiate nucleophilic 1,4‐attack akin to the Morita–Baylis–Hillman or the Rauhut–Currier reactions.[^13^, ^14^] The transiently generated enolate could then engage in polar aryl migration via a Meisenheimer intermediate with subsequent loss of SO_2_. The resulting carboxamide anion would then be expected to undergo proton exchange and eliminate the Lewis base, liberating the desired alkene product.

However, this plan was not without its possible caveats: for instance, the possibility that the presence of Lewis base might lead to competing Rauhut–Currier‐type dimerisation or amide deacylation at rates comparable to those of 1,4‐addition, with the potential dangers resulting from the liberation of a sulfonamide in solution, which would be a setback. It was also unclear whether the product, still a competent Michael acceptor, might outcompete the substrate for addition by the Lewis base, possibly leading to further undesired products.

We started our investigations with a screening of reaction conditions using *N‐*acryl‐*N*‐sulfonylimide **1 a**, bearing an electron‐deficient *p*‐nitrophenyl group, in combination with several Lewis bases. These experiments showed that 1,4‐diazabicyclo[2.2.2]octane (DABCO) affords the highest yield of α‐aryl acrylamide **2 a** (Table 1, entry 1, see Supporting Information for further details). The major side‐products found were, as foreseen, a Rauhut–Currier‐type dimerisation product **1‐rc** and the 1,4‐addition product **1‐sa** presumably generated via in situ release of sulfonamide, resulting from DABCO‐mediated deacylation. Addition of alcohols to the mixture to potentially mediate proton transfer or switching to other solvents did not increase the yield of **2 a** (entries 2–4). To decrease the extent of the Rauhut–Currier‐type side reaction, we lowered the overall concentration, which had a substantial impact on the reaction outcome (entries 5, 6). Encouraged by the high yield under dilute conditions and with longer reaction time, we sought to decrease DABCO loading, as in principle, only catalytic amounts should be required due to its release at the end of the reaction (Scheme 1F). In the event, a substoichiometric amount of DABCO afforded an 80 % yield at 80 °C, offering a quasi‐catalytic protocol variant.^[15]^ We attribute the need for larger amounts of DABCO to the formation of a DABCO‐(SO_2_)_2_ adduct, often referred to as DABSO,^[16]^ which was observed to precipitate throughout the reaction.

**Table 1:** Optimisation of the reaction conditions.

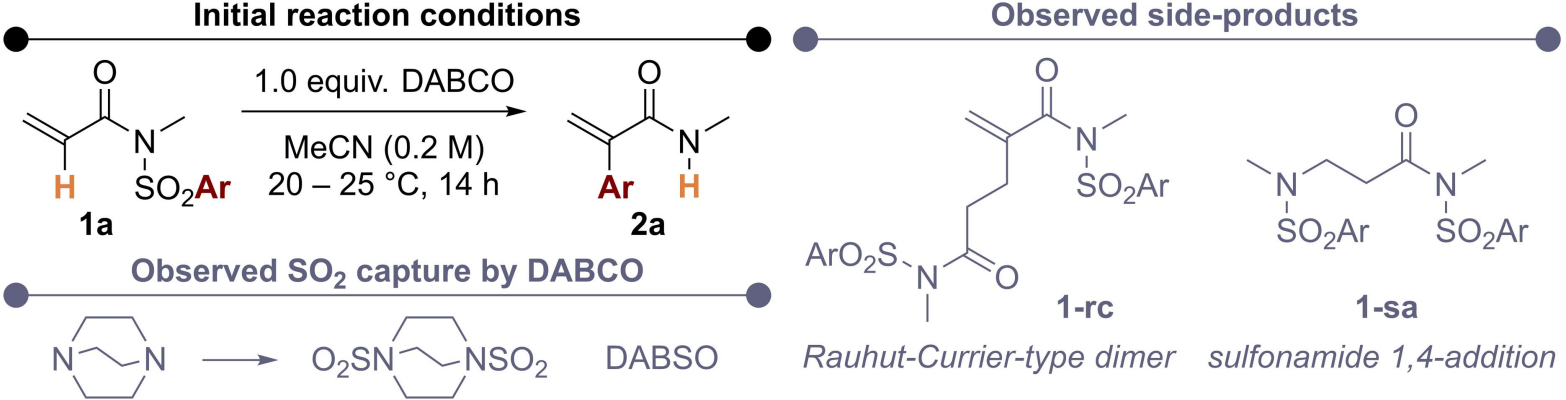				
Entry	Deviation in conditions	**2 a^[a]^**	**1‐rc^[a]^**	**1‐sa^[a]^**
1	none	40 %	5 %	4 %
2	additive: 1.0 equiv. BnOH	30 %	3 %	2 %
3	solvent: MeCN:^t^BuOH 9 : 1	30 %	3 %	2 %
4	solvent: DMF	20 %	<1 %	n.d.^[b]^
5	0.05 M	63 %	1 %	n.d.^[b]^
6	**0.05 M; 72 h**	**94 %**	**3 %**	**2 %**

Ar=*p‐*NO_2_−C_6_H_4_. [a] Yield determined by ^1^H NMR spectroscopy using mesitylene as an internal standard. [b] Not determined due to peak overlap.

For subsequent investigations of the scope, we chose to employ the room‐temperature protocol (Scheme 2). The steric requirements of the *N*‐substituent were probed with *iso*‐propyl (**2 b**) and *tert*‐butyl (**2 c**) groups, leading to a minor decrease in yield. Allyl (**2 d**) and benzyl (**2 e**) substituents, as well as a remote acetal (**2 f**) were tolerated under the reaction conditions. Several derivatives of bioactive compounds, such as amino esters (**2 g** and **2 h**), as well as dopamine (**2 i**) and tryptamine (**2 j**), showcased the broad applicability of the method. These examples also provide information about the tolerance for functional groups such as ester (**2 g**), sulfide (**2 h**) and carbamate (**2 j**), which all remained untouched. A tethered α,β‐unsaturated ester also did not interfere, even at 80 °C, yielding amide **2 k** in 81 % yield.

**Scheme 2:**
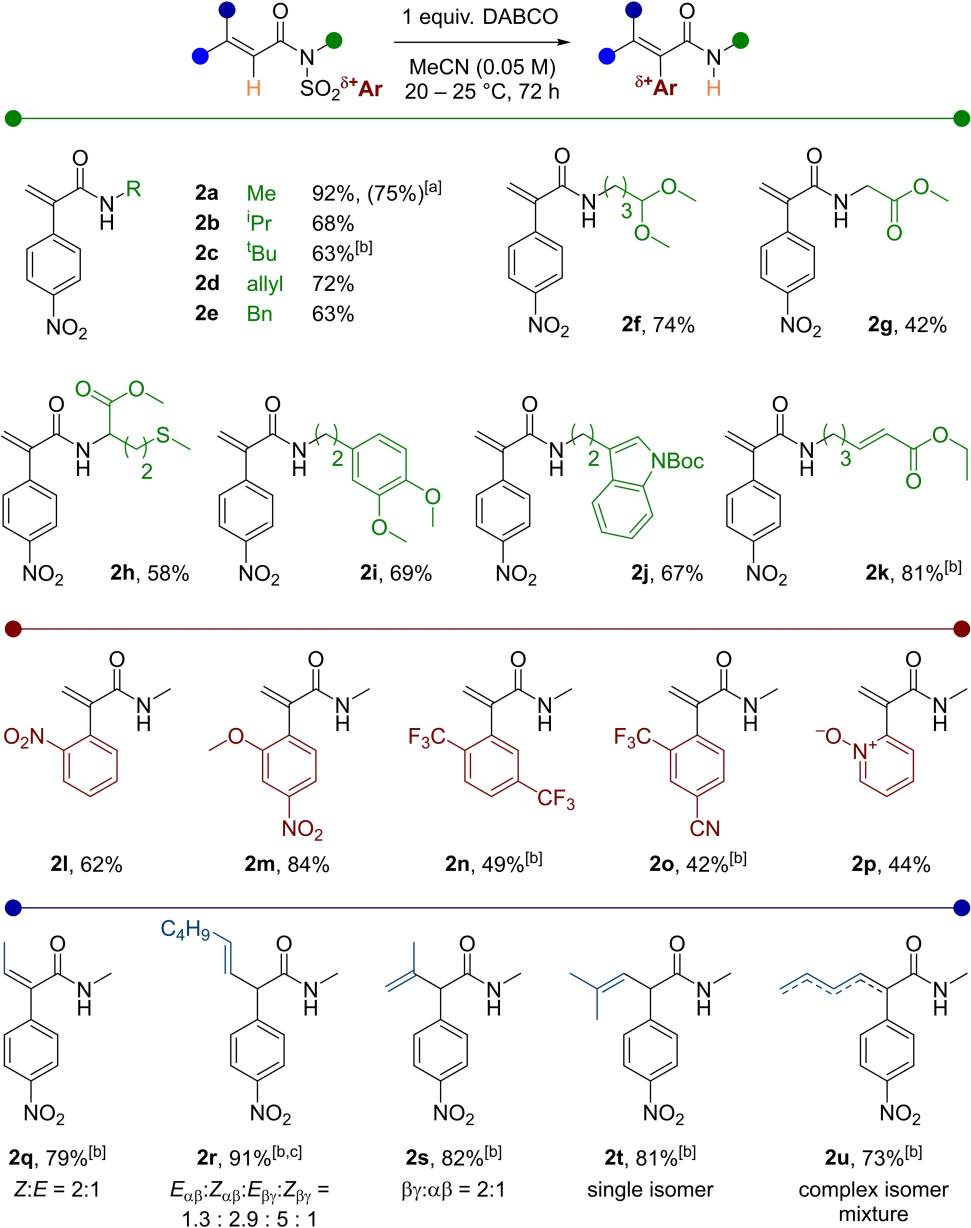
The reaction scope in regard to different substitution patterns on a 0.2 mmol scale. In case of multiple alkene isomers, the major one is showcased and the ratio given below. [a] Performed on 1 mmol scale. [b] Performed at 80 °C. [c] Performed for 120 h.

The reaction is specific for electron‐deficient *N*‐aryl sulfonamides, including the *ortho*‐isomer of **2 a** (**2 l**). The strong inductive effect of the nitro group allowed the concomitant introduction of an electron‐donating methoxy moiety (**2 m**). Arenes bearing two trifluoromethyl groups (**2 n**), as well as a trifluoromethyl group, and a nitrile (**2 o**) are also amenable substrates. In addition, an electron‐deficient heteroarene was also found to rearrange to the desired 2‐vinylpyridine *N*‐oxide (**2 p**) in moderate yield.

The method is not limited to β‐unsubstituted acrylimides, although β‐alkyl substrates require higher temperatures to engage in this rearrangement. Both β‐methyl‐ (**2 q**) and β‐*n*‐pentyl (**2 r**) substrates delivered the products in high yields. Importantly, the latter substrate preferentially formed a β,γ‐unsaturated amide product. This selectivity was also observed with β,β‐dimethyl acrylamide **2 s** and most clearly in **2 t**. Finally, an α,β,γ,δ‐conjugated diene led to formation of a mixture of aryl‐rearranged isomers **2 u** in good yield. For additional scope entries, showing the limitations of this method, see Supporting Information.

Intrigued by the elimination selectivity of **2 r**, we performed the reaction using a strong, non‐nucleophilic guanidine base (Barton's base) instead of DABCO. As shown in Scheme 3A, the Brønsted base delivers the internal alkene isomer ***iso*** **‐2 r**, albeit in lower yield.

**Scheme 3:**
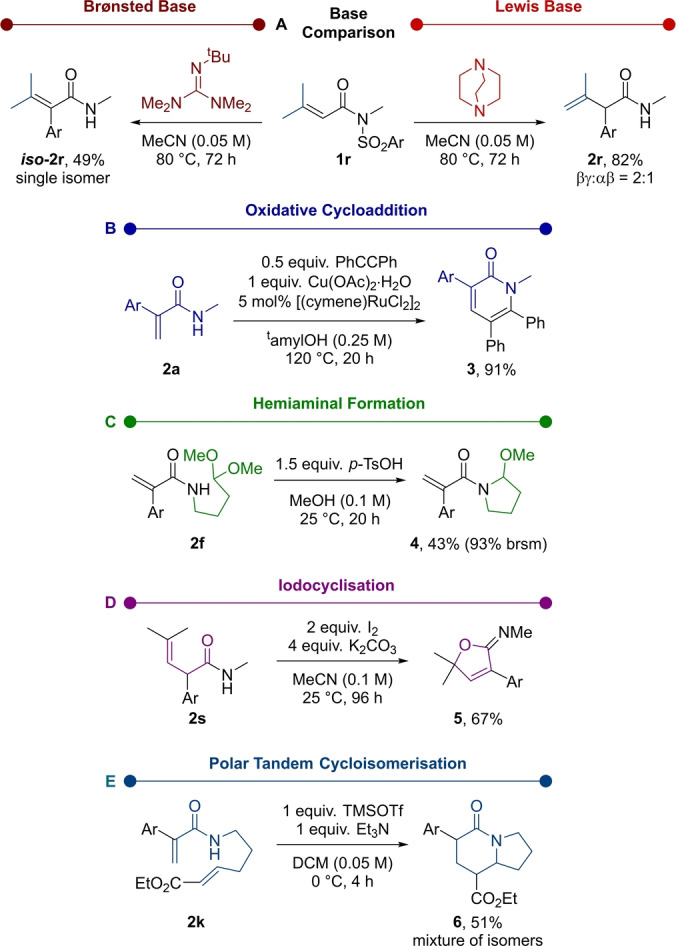
Ar=*p*NO_2_−C_6_H_4_. A) Comparison of selectivity between a Brønsted and a Lewis base. B–E) Further reactions employing the newly synthesised α‐aryl amides.

Experiments showcasing the synthetic utility of the formed α‐aryl acrylamides are depicted in Scheme 3B–E. Ruthenium‐catalysed oxidative cycloaddition of acrylamide **2 a** and diphenyl acetylene, using conditions reported by Ackermann, led to pyridone **3** in high yield.^[1d]^ Additionally, acid‐catalysed hemiaminal formation on **4** proceeded smoothly,^[17]^ and iodocyclisation/elimination of β,γ‐unsaturated amide **2 s** led to imidate **5** in good yield.

Finally, a polar tandem cycloisomerisation via an aza‐1,4‐addition, followed by a Michael addition of bis‐acryl product **2 k**, smoothly delivered aryl 2‐oxo‐octahydroindolizine **6**, showcasing an example for the possibility of rapid increase in compound complexity starting from α‐aryl acrylamides.^[18]^

To shed some light on the mechanism, quantum chemical calculations were performed at the density functional theory (DFT) level (Figure 1, see Supporting Information for further details). The obtained energy profile starts from the separated reagents, DABCO and substrate **1 a**, which form the reactant complex **A**. The first step is a kinetically favourable and slightly endergonic aza‐Michael addition, leading to the betaine intermediate **B** (Δ*G* ^≠^(**1 a→B**)=13.2 kcal mol^−1^). The following step is a Truce‐Smiles rearrangement, step **B→C**, which involves a concerted asynchronous C−C bond formation and C−S bond cleavage via an early “Meisenheimer transition state” **TS_BC_**.^[19]^ This step presents a barrier which is only 0.8 kcal mol^−1^ higher than the reverse Michael addition (step **B→A**), but the high exergonicity of this Truce‐Smiles rearrangement (Δ*G*(**B→C**)=−31.1 kcal mol^−1^) irreversibly drives the reaction towards the formation of intermediate **C**. In contrast to initial reaction design, direct SO_2_ release or SO_2_ transfer processes from **C** were shown to be energetically prohibited.^[20]^ However, we found that enolisation of **C** was accessible through the action of a second molecule of DABCO, which can deprotonate the α‐position of the carbonyl (Δ*G* ^≠^(**C→E**)=24.3 kcal mol^−1^) to form intermediate **E**, where the negative charge is highly delocalised in the aromatic core (see Supporting Information). The subsequent *E1cb* elimination involves the cleavage of the N−C bond to release DABCO (Δ*G* ^≠^(**D→E**)=8.2 kcal mol^−1^), yielding the ammonium sulfinate **F** that is nearly isoenergetic to **E**. The last step is an exergonic DABCO‐mediated SO_2_ transfer (Δ*G*(**F→G**)=−7.9 kcal mol^−1^) with concomitant nitrogen atom protonation and DABCO nucleophilic substitution at sulfur.^[21]^ The transition state for this step, **TS_FG_**, lies only 0.7 kcal mol^−1^ above the transition state of the previous step, **TS_EF_**; therefore the formation of the product is driven by the exergonicity of this final step. This last, thermodynamically favoured, SO_2_ transfer thus generates the product **2 a** through protonation, along with a (DABCO)_2_⋅SO_2_ species finding stabilisation through hydrogen bonding as shown in **G**. Indeed, such a proton transfer sequence was supported by an experiment using deuterium labeled **1 a** (see Supporting Information for further details). Furthermore, (DABCO)_2_⋅SO_2_ ultimately converges to the formation of the more thermodynamically stable DABSO species (Δ*G*(**(DABCO)_2_** ⋅**SO_2_→DABSO**)=−12.3 kcal mol^−1^) during the reaction, which then precipitates as observed empirically.

**Figure 1:**
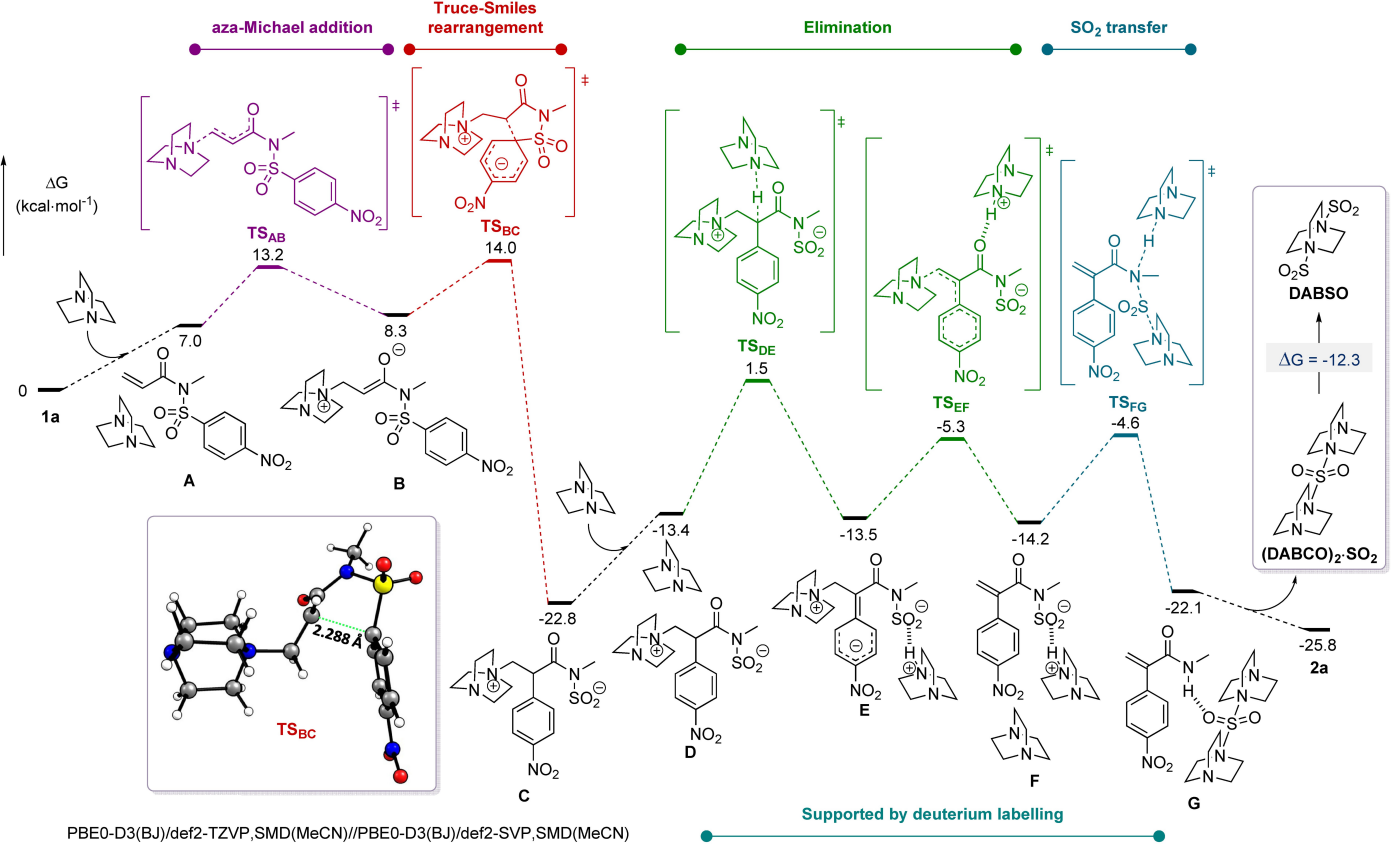
Computed reaction profile at the PBE0‐D3(BJ)/def2‐TZVP,SMD(MeCN)//PBE0‐D3(BJ)/def2‐SVP,SMD(MeCN) level of theory.

In conclusion, we have reported a method for the synthesis of α‐aryl acrylamides, relying on a polar aryl migration. The Lewis base DABCO mediates the process even at ambient temperature, rendering the reaction mild and allowing for a wide range of substituents and functional groups to be tolerated. Taking advantage of the reactive handles of the products and highlighting their utility, further transformations into various cyclic compounds were demonstrated. Finally, quantum chemical calculations elucidated the details of the underlying mechanism and revealed the triple role of DABCO (1,4‐addition; proton shuttle; SO_2_ transfer).

## Conflict of interest

The authors declare no conflict of interest.

## Supporting information

As a service to our authors and readers, this journal provides supporting information supplied by the authors. Such materials are peer reviewed and may be re‐organized for online delivery, but are not copy‐edited or typeset. Technical support issues arising from supporting information (other than missing files) should be addressed to the authors.

Supporting Information

## Data Availability

The data that support the findings of this study are available in the supplementary material of this article.
